# Effects of general anesthetics on seizure termination, mortality, and neurological prognosis in a rat model of pediatric refractory status epilepticus: A comparative randomized controlled study

**DOI:** 10.1016/j.jare.2025.10.076

**Published:** 2025-11-02

**Authors:** Bo Chen, Yuping Zhao, Na Shen, Feng Liu, Li Jiang, Yuanlin Ma, Xin Tian, Patrick Kwan

**Affiliations:** a Department of Anesthesiology, Chongqing University Cancer Hospital, Chongqing 400030, PR China; b Department of Geriatrics, Laboratory of Research and Translation for Geriatric Diseases, the First Affiliated Hospital of Chongqing Medical University, Chongqing 400016, PR China; c Department of Neurology, the First Affiliated Hospital of Chongqing Medical University, Chongqing Key Laboratory of Major Neurological and Mental Disorders, Chongqing 400016, PR China; d Department of Epilepsy Center, The First Affiliated Hospital of Chongqing Medical University, Chongqing 400016, PR China; e Key Laboratory of Major Brain Disease and Aging Research (Ministry of Education), Chongqing Medical University, Chongqing 400016, PR China; f Department of Critical Care Medicine, the First Affiliated Hospital of Chongqing Medical University, Chongqing 400016, PR China; g Department of Intensive Care, the Seventh People’s Hospital of Chongqing, Chongqing 400054, PR China; h Department of Rehabilitation Medicine, the First Affiliated Hospital of Chongqing Medical University, Chongqing 400016, PR China; i Department of Neurology, the Affiliated Yongchuan Hospital of Chongqing Medical University, Chongqing Key Laboratory of Cerebrovascular Disease Research, Chongqing 402160, PR China; j Department of Neuroscience, School of Translational Medicine, Monash University, Melbourne, Australia

**Keywords:** Refractory status epilepticus, Lithium-pilocarpine rat model, General anesthetics, Seizure termination, Mortality, Neurological prognosis

## Abstract

**Introduction:**

Refractory status epilepticus (RSE) is a serious neurological emergency in children associated with significant mortality and morbidity. General anesthetics (GAs) are routinely used to treat RSE. However, the optimal selection of GAs for pediatric RSE remains unknown.

**Objective:**

We aimed to identify the optimal agent by comparing the efficacy and safety of different GAs in a rat model of RSE.

**Methods:**

Sprague-Dawley rats at postnatal day 20 were subjected to lithium-pilocarpine-induced SE. Animals that developed RSE (pharmacoresistant to the first- and second-line therapies) were randomized to receive one of five GAs (midazolam, propofol, dexmedetomidine, esketamine, or sevoflurane) as the third-line therapy or vehicles. The latency to SE termination (time from the initiation of GAs to SE termination) and 24-hour mortality were assessed. On Day 5 following SE, a series of behavioral tests were performed to evaluate the behavior and cognition.

**Results:**

All five GAs effectively terminated the RSE, with propofol and sevoflurane having the shortest latencies to SE termination. GAs significantly improved survival after the complete termination of SE. Additionally, GAs reduced the cognitive deficits caused by SE, especially episodic memory, without affecting the behavioral abnormalities. Finally, we proposed a multi-dimensional model that integrated the efficacy, safety, and effect of GAs on neurological prognosis, suggesting that sevoflurane might be the optimal treatment.

**Conclusion:**

Our study showed that treatment with GAs effectively terminated RSE and improved survival and short-term cognition. Our proposed model suggests that volatile anesthetics may be the optimal choice for treating RSE. These results may be used to inform the design of comparative clinical trials of GAs for RSE in children.

## Highlights

• Optimizing general anesthetics remains an unsolved challenge in clinical refractory status epilepticus (RSE).
• Different general anesthetics have distinct benefits and risks for treating RSE.
• Five anesthetics were compared for seizure control, survival, and neurological outcomes.
• A multi-dimensional model is proposed to guide optimal anesthetic selection in RSE.
• Volatile anesthetics may be the optimal choice when balancing benefits and risks.

## Graphical abstract

**Figure:**
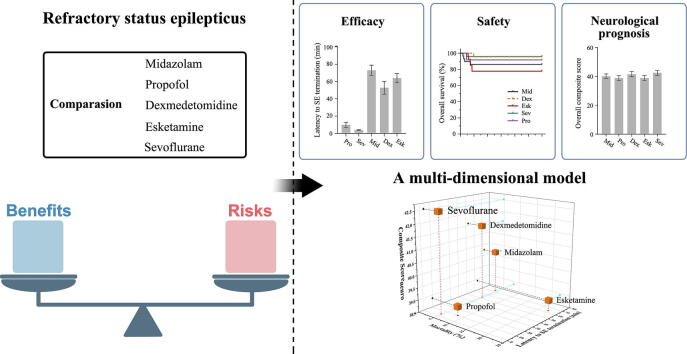

## Introduction

Convulsive status epilepticus (SE) is the most common neurological emergency in childhood, with an annual incidence of up to 42 per 100,000 children [1]. Mortality in pediatric convulsive SE is approximately 3 % in the short term [1] and 7 % to 11 % in the long term [2]. Furthermore, lifelong sequelae, including cognitive impairment, behavioral problems, and subsequent epilepsy, are common and have a major negative impact on the quality of life of the survivors [3].

Current guidelines recommend a stepwise treatment paradigm for SE, with benzodiazepines (BZDs) as the first-line therapy, followed by sequential addition of antiseizure medications (ASMs) as the second-line therapy if SE persists [4]. When SE continues despite treatment with first- and second-line agents, it is considered refractory (RSE) [5], for which aggressive treatment using general anesthetics (GAs) as the third-line therapy is recommended [6]. Commonly used GAs in this setting include midazolam, propofol, barbiturates, and ketamine [6]. However, observational studies in adults suggested that the use of GAs was associated with worse outcomes, including increased risk of death and infection [[7], [8], [9]]. These studies were limited by their retrospective nature which has inherent bias, including a tendency for patients with more severe RSE to receive GAs [5]. In addition, there is concern that early repeated exposure of the developing brain to GAs might lead to impaired neurodevelopment and long-term cognition deficits [[10], [11], [12]]. Previous prospective randomized controlled clinical trials comparing the different GAs or between GAs and non-anesthetic treatments have failed to complete owing to practical constraints such as insufficient recruitment and ethical concerns [6].

We aimed to address these knowledge gaps by conducting a randomized controlled animal study. We compared the efficacy of five different GAs in terminating RSE and their effects on mortality and neurological prognosis in a rat model of RSE. These agents are commonly used in the operating room (OR) and intensive care unit (ICU). Based on the findings, we proposed a multi-dimensional model to aid in selecting the optimal GA for RSE that integrated the efficacy, safety, and neurological prognosis.

## Material and methods

### Animals

Pregnant Sprague-Dawley rats were housed in individual cages in a 12-hour light/dark cycle with food and water *ad libitum*. After delivery, litters were adjusted to 12 pups for homogeneity, and the day of birth was recorded as postnatal day 0 (PND 0). Pups were weaned after electroencephalography (EEG) electrode implantation on PND16. Experiments were approved by the Ethics Committee of Chongqing University Cancer Hospital (approval number: CZLS2023085-A-47) and performed in accordance with the National Institutes of Health Guidelines for the Care and Use of Laboratory Animals (8th edition, 2011).

### Drugs

Lithium chloride, pilocarpine hydrochloride, and scopolamine methyl bromide were dissolved in distilled water to concentrations of 10, 5, and 0.1 mg/mL, respectively. Phenytoin sodium was dissolved at 5 mg/mL in a vehicle of 40 % propylene glycol, 10 % ethanol, and 1.5 % benzyl alcohol. The injection volume was minimized (< 0.2 mL) to control for potential vehicle effects, and our separate preliminary experiments confirmed the absence of intrinsic anticonvulsant activity in this vehicle alone. Diazepam (5 mg/mL) was diluted to 0.5 mg/mL in 5 % glucose saline (GS). Midazolam (5 mg/mL) and dexmedetomidine (200 μg/mL) were diluted in normal saline (NS) to 1 mg/mL and 4 μg/mL, respectively. Esketamine was dissolved in NS to 2 mg/mL. Propofol (10 mg/mL) was diluted in intralipid to a concentration of 1 mg/mL. All drugs were administered by intraperitoneal (*i.p.*) injection except sevoflurane, which was delivered by inhalation. The detailed information on drugs is listed in the Supplementary Table 1.

### Electrodes implantation and EEG recordings

Rats were anesthetized with sevoflurane and implanted with the cortical electrodes. EEG recordings were visually examined during SE and analyzed offline to calculate the power spectral density (PSD). For details, see the Supplementary Methods.

### Experimental design and outcomes analysis

**Fig. 1:**
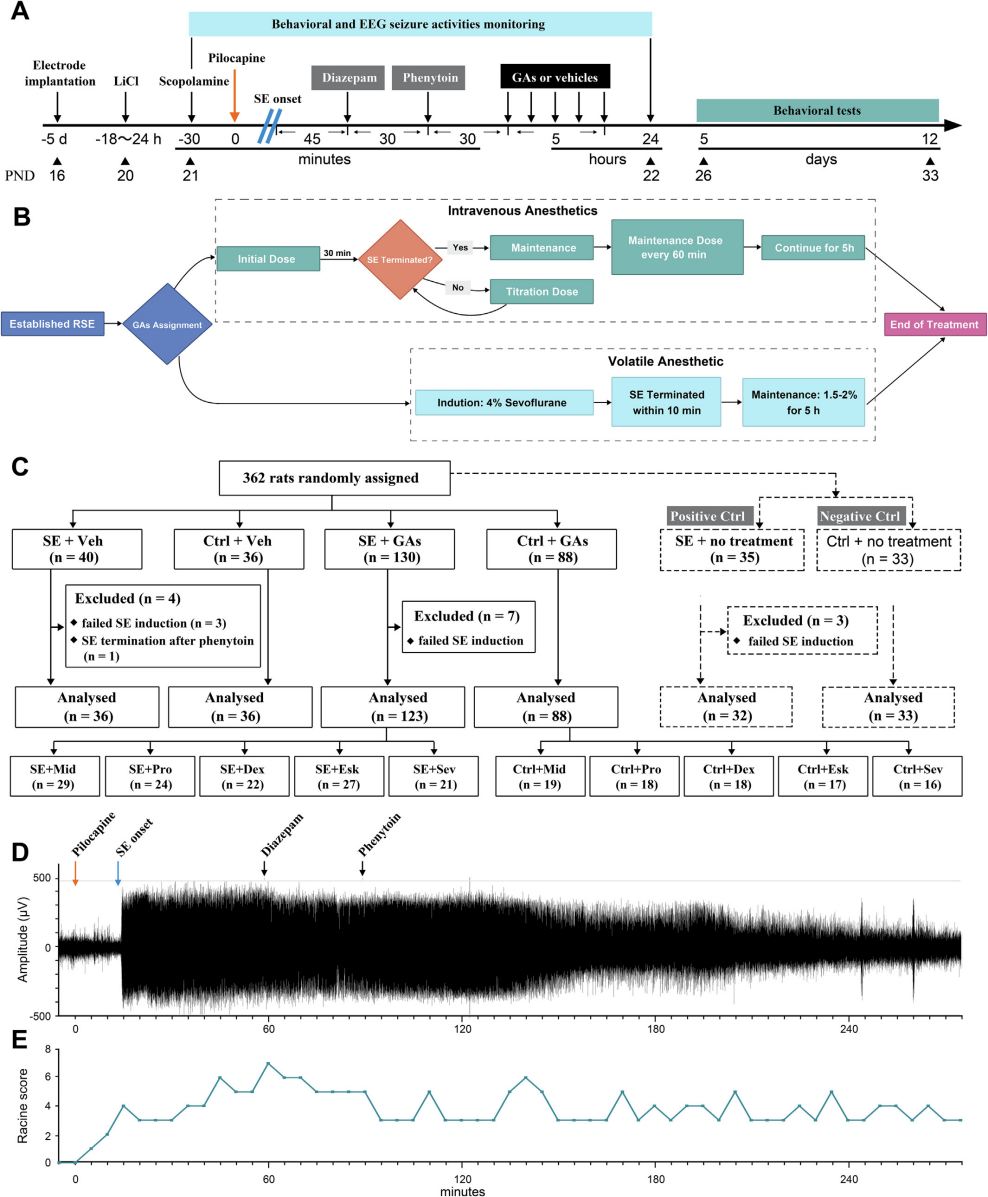
The experimental design and an animal model of refractory status epilepticus. (A) Experimental paradigm. (B) Flowchart of the conditional dosing protocol for GAs. (C) Flowchart of the animals inclusion. An animal model of refractory status epilepticus was developed by delayed administrations of antiseizure medications and confirmed by (D) a representative EEG recording of sustained seizure activity and (E) corresponding behavioral seizure activity in one rat after treatment with diazepam and phenytoin in the model of SE. Each dot is the maximum Racine score within the 5-minute interval. PND, postnatal day; Ctrl, control; Veh, vehicle; Mid, midazolam; Pro, propofol; Dex; dexmedetomidine; Esk, esketamine; Sev, sevoflurane.

The overall experimental protocol is illustrated in Fig. 1A. The animal model of SE was established by systemic administration of lithium-pilocarpine. Similar to the clinical features of SE, animals subjected to experimental SE present with general convulsions, EEG abnormalities, and high mortality. Surviving animals show cognitive impairment and neurodegenerative pathology. Additionally, when treatment is delayed, SE becomes refractory to BZDs and other ASMs [13,14].

In this study, the first-line (diazepam) therapy was initiated 45 min following the onset of SE since refractoriness to diazepam at this time point has been previously confirmed [14]. The second-line (phenytoin) therapy was given 30 min after diazepam administration. Rats that displayed persistent behavioral and electrographic SE for a further 30 min were defined as having RSE and given a GA as the third-line therapy. GAs were initially titrated to achieve the goal of SE termination, and then administered repeatedly to maintain the seizure-free status. Mortality within 24 h after SE, SE duration (time from the onset of SE to SE termination), and latency to SE termination (time from the initiation of GAs to SE termination) were recorded. After recovery of SE (5 days), neurological outcomes were assessed by a series of behavioral tests in the surviving rats.

Rats were randomly assigned to six main groups (Fig. 1C): SE + GAs, SE + Veh, Ctrl + GAs, Ctrl + Veh, SE + no treatment, and Ctrl + no treatment. Rats in the SE + GAs group were further randomized into 5 subgroups, each receiving one of the five GAs, namely midazolam, propofol, dexmedetomidine, esketamine, and sevoflurane. Rats in the SE + Veh group received an equal volume of vehicles. Rats in the Ctrl + GAs group (no SE) received saline instead of pilocarpine and were further randomized into 5 groups, each receiving one of the five GAs, similar to the SE + GAs group. Rats in the Ctrl + Veh group (no SE) received saline instead of pilocarpine followed by vehicle. Rats in the SE + GAs, SE + Veh, Ctrl + GAs, and Ctrl + Veh groups received administrations of diazepam and phenytoin before receiving GA or vehicle. Rats in the SE + no treatment and Ctrl + no treatment (no SE) groups did not receive any treatment with ASMs or GAs and acted as the positive and negative controls for the study, respectively. The numbers of male and female rats in each group were shown in Supplementary Tables 2 and 3.

### Induction of SE

SE was induced by pretreatment with lithium chloride (3 mEq/kg) on PND 20 followed 18–24 h later by pilocarpine hydrochloride (60 mg/kg). Scopolamine methyl bromide (1 mg/kg) was administered 30 min before pilocarpine to decrease peripheral cholinergic effects. Following pilocarpine injection, both behavioral performance and EEG signal were continuously monitored in real-time by an experienced experimenter blinded to the treatment condition. Behavioral seizure activity was scored using a modified extended Racine scale[15]: stage 1: a sudden change in behavioral state; stage 2: head nodding; stage 3: forelimb clonus; stage 4: rearing, bucking, or clonus when lying on belly; stage 5: falling or clonus when lying on the side; stage 6: multiple sequences of rearing and falling, or brief jumping; stage 7: violent jumping; stage 8: violent jumping or wild running followed by a period of tonus lasting longer than 5 s. Electrographic seizure activity was defined as the rhythmic high-amplitude spikes (>3 x baseline) that lasted at least 10 s [16]. The onset of SE was determined by the EEG signal and behavioral scoring of seizure activity. The first clonic seizure (a Racine stage 3), accompanied by 30 s continuous rhythmic epileptiform activity on EEG was taken as the onset of SE [17]. Only rats displaying sustained behavioral and electrographic seizure activities were used in the following experiments.

### Treatments of SE with ASMs

Forty-five minutes after the onset of SE, diazepam (5 mg/kg) was administered, and its pharmacoresistance was established 30 min later by ongoing behavioral and electrographic SE, which is according to the protocol of the experimental RSE [13, 14. To more realistically simulate the clinical RSE, phenytoin (25 mg/kg) was injected 30 min after the diazepam administration. Another 30 min later, rats with continuous SE were defined as having RSE and treated with GAs or vehicles. The selection of diazepam and phenytoin as the first- and second-line ASMs was based on the clinical guideline [4] and established experimental models of RSE [14,18]. The doses of diazepam and phenytoin were chosen on the basis that they have been shown to achieve therapeutic concentrations in the immature brain [14,19].

### Treatments of SE with GAs

The GAs administered included the intravenous anesthetics (IAs) midazolam, propofol, esketamine, and dexmedetomidine, and the volatile anesthetic (VA) sevoflurane. The conditional dosing protocol is depicted in the flowchart of Fig. 1B. A series of pilot studies were carried out to determine the administration procedures and dosages of four IAs. We found that a single anesthesia dose (loss of righting reflex and no response to tail-clamp stimulation) of IAs was required to terminate SE in the SE + GAs group. However, this single high dose of IAs caused severe respiratory suppression and hypoxia, with 70 % of rats dying due to respiratory arrest within 30 min after IAs administration. Therefore, we decided to control SE at a relatively low initial dose of IAs to induce sedation without apnea and titrated a low dose of IAs at 30-minute intervals until SE was terminated. Initial and titration doses were as follows: midazolam: 18 mg/kg and 6 mg/kg; propofol: 20 mg/kg and 5 mg/kg; esketamine: 40 mg/kg and 10 mg/kg; dexmedetomidine: 60 μg/kg and 20 μ g/kg. SE termination was defined as complete cessation of behavioral seizures (stage < 2), and the EEG activity returned to baseline after the last injection of GAs [16]. The time of SE termination was recorded as the point at which anesthesia began to produce efficacy. Subsequently, repeated injections of midazolam, propofol, esketamine, and dexmedetomidine at doses of 9 mg/kg, 10 mg/kg, 20 mg/kg, and 30 μ g/kg, respectively, were given every 60 min to maintain the seizure-free status for 5 h. Rats in the Ctrl + GAs group received the same doses and frequency of administration. All initial, titration, and maintenance doses described above were determined based on our pilot study results as well as previously published research [[20], [21], [22], [23]].

For rats in the SE + GAs sevoflurane subgroup, anesthesia was induced with 4 % sevoflurane in 90 % oxygen (1 L/min) via a face mask. When SE termination was achieved, sevoflurane concentration was maintained at 1.5–2 % for 5 h. Rats in the Ctrl + GAs sevoflurane subgroup received the same concentration of sevoflurane for anesthesia induction, but the induction duration was limited to 3 min, which was sufficient to achieve the level of deep anesthesia. Then, 1.5–2 % of sevoflurane was delivered via a face mask for 5 h. The duration of anesthesia was determined based on the results of our pilot study, in which SE lasted for a maximum of 5 h in the SE + Veh group. The sevoflurane dosing regimen was established based on evidence from our pilot study and published research [24].

During the GAs exposure, all rats breathed spontaneously and were supplied continuous oxygen using the face mask. Oxygen saturation was monitored using pulse oximetry, and rectal temperature was monitored and maintained at 37 ± 1 ℃ using a heating blanket. Animals in the SE + Veh and Ctrl + Veh groups received injections of vehicles at equivalent volumes and frequency. After 5 h of GAs exposure, four rats from each group were used for arterial blood gas analysis. The blood samples were drawn from the left femoral artery and analyzed immediately using the GEM Premier 5000 blood-gas analyzer (Instrumentation Laboratory, Bedford, MA, USA).

### Behavioral tests

Five days after SE, open field (OF), object recognition memory (ORM), and Morris water maze (MWM) were performed in a sound-attenuated room with controlled light intensity (3 lx) using the methods we described previously [25]. For details, see the Supplementary Methods.

### Composite scores for outcomes of behavioral tests

A composite score system was used to summarize multiple outcomes of behavioral tests as described previously [26]. For details, see the Supplementary Methods.

### Statistical analysis

Statistics were performed using SPSS (IBM, NY, USA), GraphPad Prism (GraphPad Software Inc., CA, USA), and OriginPro (OriginLab, MA, USA) software. Data were presented as the means ± standard deviation (SD) or median (interquartile range). A significant difference was considered at *p* < 0.05. For behavioral tests in lithium-pilocarpine-treated rats, the minimal sample size was determined by preliminary data from the ORM test, which showed a power of more than 85 % with n = 15 per group. For comparing differences in efficacy and mortality in GAs-treated rats, sample sizes were not predetermined but were similar to those previously reported in the literature [27].

Normality and homogeneity of variance were detected using Kolmogorov-Smirnov and Levene’s tests, respectively. Two-way analysis of variance (ANOVA) or two-way repeated ANOVA was used to assess the normally distributed data, as appropriate, followed by Sidak’s *post hoc* analysis. For nonparametric outcomes, the Scheirer-Ray-Hare test or Kruskal-Wallis test followed by Dunn’s multiple comparisons was performed. Survival curves were plotted using the Kaplan-Meier method, and differences between groups were compared using a log-rank test. To address the time-dependent nature of differences in survival, a landmark analysis was performed on the survival data. All tests were two-sided.

The median effective dose (ED_50_) for SE termination was calculated for each IA using survival analysis. This approach applies a “dose-to-event” paradigm, where the cumulative dose at which SE terminated was analyzed as the primary variable, and the event was defined as successful termination with animal survival. Animals that died from GA-related complications were censored at the dose of death. The ED_50_ value and its 95 % confidence interval (CI) were derived from the Kaplan-Meier analysis. To evaluate the rationale of the initial dosing strategy, the percentage of the ED_50_ covered by the initial dose was calculated for each IV as: (Initial Dose / ED_50_) x 100 %.

## Results

### Establishment of a model of RSE

**Fig. 2:**
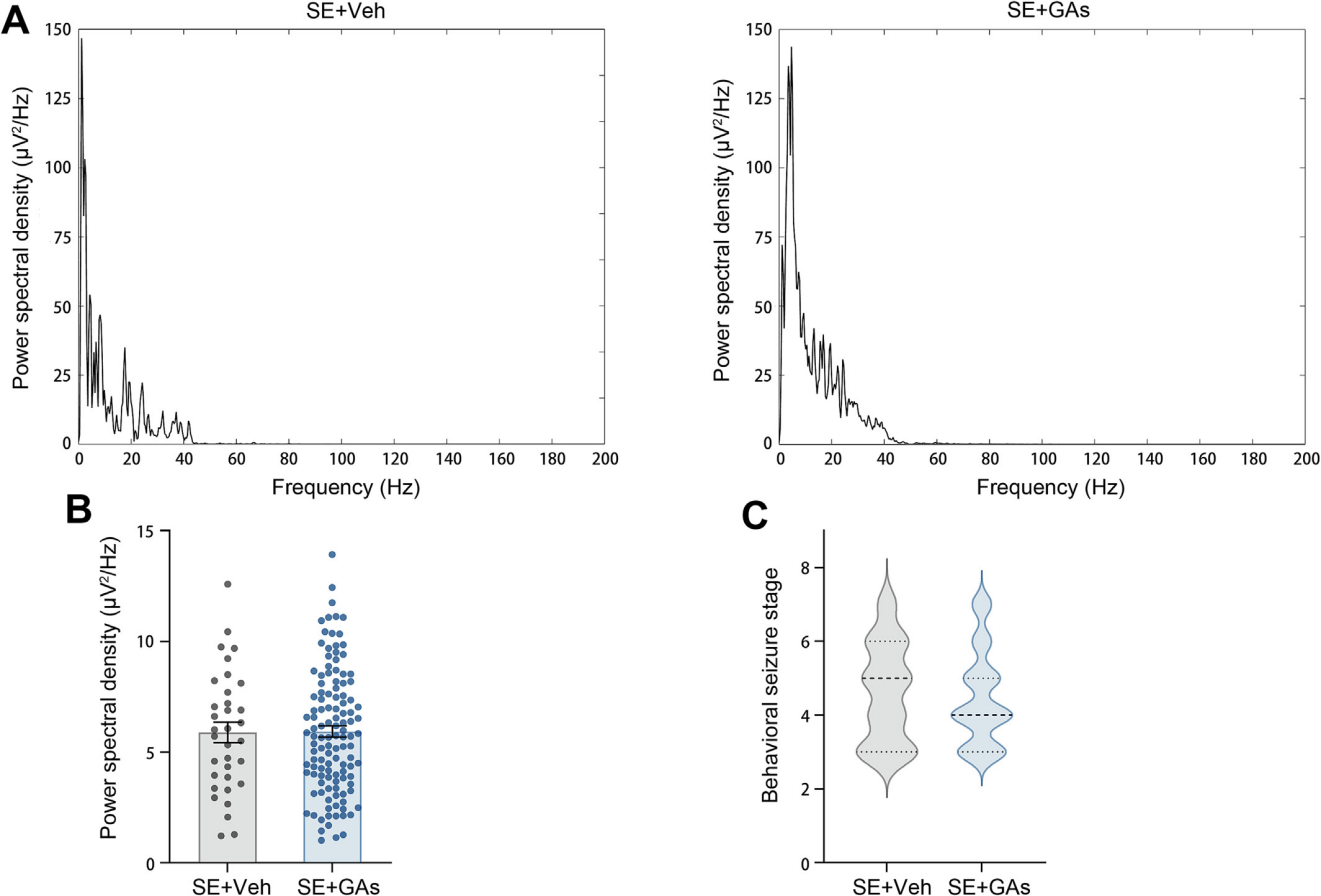
SE severity in the rats treated with GAs or vehicles. (A) The mean Fast Fourier Transform over 1-min EEG recordings before treatment with vehicles (the left panel) or GAs (the right panel). (B) The total EEG power spectral density and (C) Racine score within 1 min before the initial injection of GAs or vehicles. Veh, vehicle. SE + Veh, n = 34; SE + GAs, n = 120. Error bars represent the standard error of the mean (SEM). Data were statistically analyzed by a two-way ANOVA followed by a Sidak’s test in the total EEG power spectral density and by the Scheirer-Ray-Hare test in the behavioral seizure activity. Power spectral density: two-way-ANOVA_sex x group_*F*_(1,150)_ = 0.05, *p* = 0.822; two-way-ANOVA_group_*F*_(1,150)_ = 0.06, *p* = 0.814; two-way-ANOVA_sex_*F*_(1,150)_ = 2.37, *p* = 0.126. Behavioral seizure stage: two-way-ANOVA_sex x group_*F*_(1,150)_ = 0.56, *p* = 0.457; two-way-ANOVA_group_*F*_(1,150)_ = 0.14, *p* = 0.712; two-way-ANOVA_sex_*F*_(1,50)_ = 0.03, *p* = 0.870.

Consistent with previous studies [14], delayed treatment with a sedative dose of diazepam did not terminate SE in any rat in this study. Phenytoin was also ineffective when administered 30 min after diazepam injection. Overall, 99.4 % (159/160) of rats that received phenytoin injection continued to experience SE. Behaviorally, the severity of seizure activity was reduced, especially the frequency of generalized tonic-clonic seizures. Rats exhibited, for most of the time, stage 2 seizures with brief intervals of rearing, falling, jumping, or wild running (Fig. 1E). Electrographically, treatment with diazepam followed by phenytoin attenuated the frequency of typical high-frequency and high-amplitude spikes but did not terminate electrographic SE, and paroxysmal epileptiform activities were still detected on EEG (Fig. 1D). In addition, SE lasted for approximately 3 h (range of 2.5–5 h) after phenytoin administration. There were no significant differences in the Racine scale or the total EEG PSD at the beginning of GAs or vehicles administration (Fig. 2A-C), indicating that SE severity was similar in the SE + GAs and SE + Veh groups.

### Efficacy of GAs on SE termination

SE without any treatments lasted for more than 8 h (548.47 ± 9.67 min), whereas SE duration either in the SE + Veh or SE + GAs group was significantly decreased (Table 1). Treatment with GAs significantly shortened the latency to SE termination compared to vehicles (Table 1). There were no differences in SE duration and latency to SE termination between male and female rats (Supplementary Fig. 2A and B).

**Table 1:** The therapeutic effect of GAs on SE.

Experimental group	SE duration (min)	Latency to SE termination (min)
Ctrl + no treatment (n = 33)	0	−
SE + no treatment (n = 19)	548.47 ± 42.17^***^	−
Ctrl + Veh (n = 36)	0	0
SE + Veh (n = 31)	241.16 ± 65.94^***,###^	136.16 ± 65.94
Ctrl + GAs (n = 86)	0	0
SE + GAs (n = 109)	146.71 ± 36.85^***,###^	41.71 ± 36.85^&&&^

Ctrl, control; Veh, vehicle; GAs. Data are statistically analyzed by a two-way ANOVA with experimental group and sex as the main effects. SE duration: two-way-ANOVA_group_*F*_(5,302)_ = 1212.12, *p* < 0.0001; two-way-ANOVA_sex x group_*F*_(5,302)_ = 0.47, *p* = 0.802. Latency to SE termination: two-way-ANOVA_group_*F*_(3,254)_ = 629.87, *p* < 0.0001; two-way-ANOVA_sex x group_*F*_(3,254)_ = 0.64, *p* = 0.591. ^***^p < 0.0001 compared with Ctrl + no treatment group; ^###^*p* < 0.0001 compared with SE + no treatment group; ^&&&^*p* < 0.0001 compared with SE + Veh group.

**Fig. 3:**
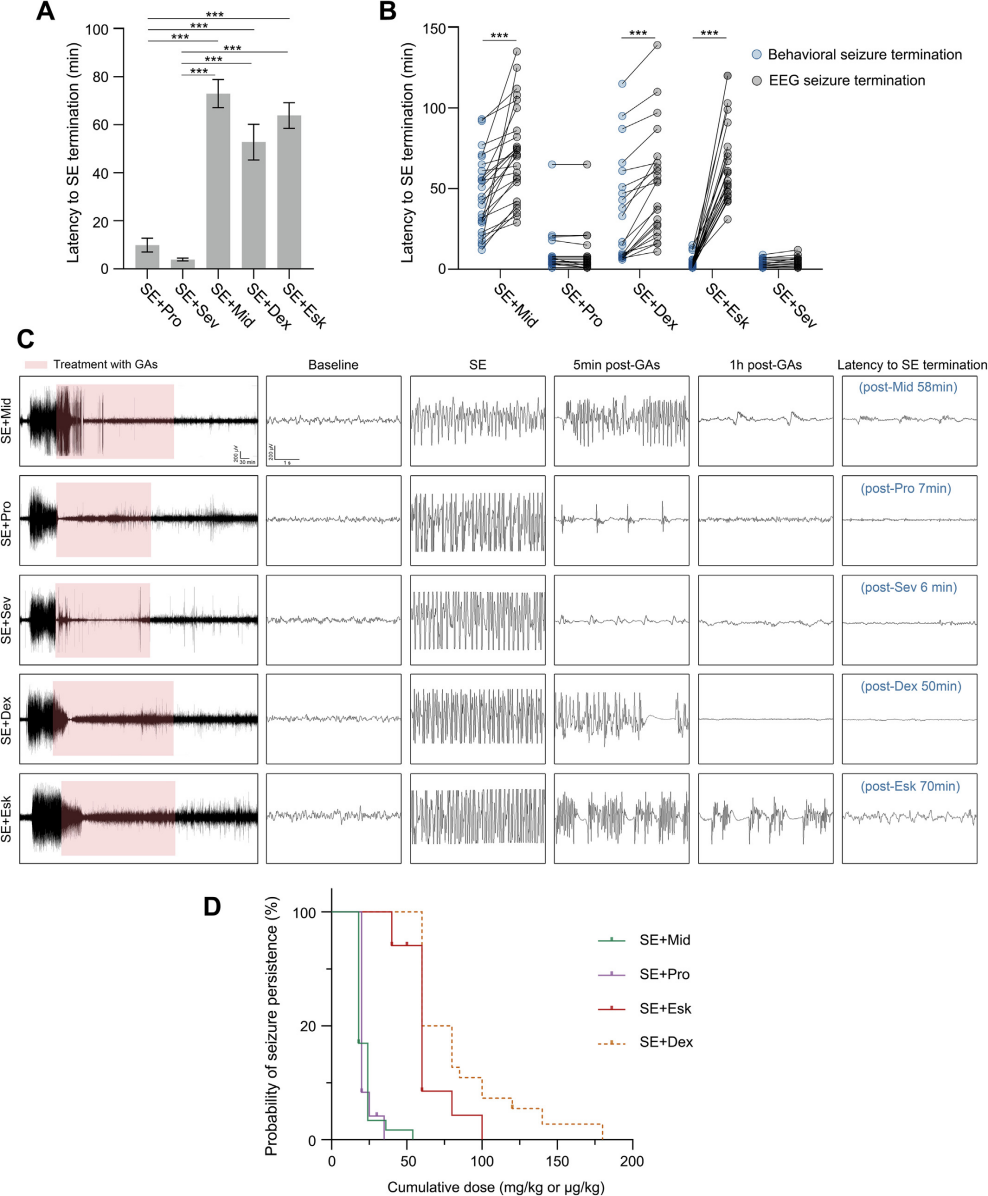
The efficacy of GAs on SE termination. (A) Effects of different GAs on electrographic SE termination represented by latency to SE termination. (B) The difference in latency between behavioral seizure and electrographic seizure termination after treatment with GAs. (C) Representative compressed EEG for an 8-h period (the left panels) and EEG tracings (the right panels) from 5 rats treated with midazolam, propofol, sevoflurane, dexmedetomidine, and esketamine, respectively, at baseline, during SE, 5 min and 1 h after GAs administration, as well as the time of SE termination. (D) Cumulative dose–response survival curves for SE termination by four IAs. Mid, midazolam; Pro, propofol; Dex, dexmedetomidine; Esk, esketamine; Sev, sevoflurane. SE + Mid, n = 25; SE + Pro, n = 22; SE + Sev, n = 20; SE + Dex, n = 21; SE + Esk, n = 21. Error bars represent the standard error of the mean (SEM). ^***^p < 0.0001. Data were statistically analyzed by a two-way ANOVA followed by a Sidak’s test for comparing the latency to SE termination of different GAs. Two-way-ANOVA_sex x group_*F*_(4,99)_ = 0.33, *p* = 0.855; two-way-ANOVA_group_*F*_(4,99)_ = 35.70, *p* < 0.0001; two-way-ANOVA_sex_*F*_(1,99)_ = 0.74, *p* = 0.391. Data were statistically analyzed by a paired *t* test for comparing the difference in latency to SE termination between behavioral and EEG seizure termination.

An initial dose of propofol and sevoflurane immediately reduced the amplitude of spikes and restored the EEG activity to baseline within 9.91 ± 2.89 and 3.85 ± 0.58 min, respectively (Fig. 3A). The cessation of behavioral seizure activity coincided with the termination of electrographic seizure activity (Fig. 3B). In comparison, the other three GAs tended to take longer to stop SE after a single initial dose (Fig. 3A). In some animals, midazolam, dexmedetomidine, or esketamine needed to be titrated several times to achieve the desired therapeutic effect (Supplementary Table 4). Further, the termination of electrographic SE lagged behind the cessation of behavioral seizure activity after treatment with these three GAs (Fig. 3B), particularly for esketamine. The frequency of epileptic spikes decreased over time, whereas the amplitude did not change significantly in the short term (Fig. 3C).

To determine the dose–response relationship for SE termination independent of the dosing regimen, we calculated ED_50_ for each IA using a dose-to-event survival analysis (Table 2). The corresponding Kaplan-Meier curves revealed that the dose requirements for propofol and midazolam were highly consistent across animals, as indicated by their steep slopes. In contrast, the more gradual declines of the curves for esketamine and dexmedetomidine indicated substantially greater inter-individual variability in the effective dose (Fig. 3D). To control for the confounding effect of the initial dose, we established the “relative initial intensity” (initial dose/ED_50_) as a standardized metric for cross-IA evaluation (Table 3). We found that for three IAs (midazolam, propofol, and dexmedetomidine), the initial dose matched the ED_50_, representing a fully theoretical dose. Conversely, the initial dose of esketamine covered only 66.7 % of its ED_50_. However, despite its inefficacy in terminating SE, the initial dose of esketamine reliably induced deep sedation or general anesthesia, an effect confirmed in control animals. We adopted a pattern of multiple repeated administrations of GAs to maintain the seizure-free status for 5 h. Thus, no SE recurrence was detected during the recovery period after anesthesia withdrawal (Fig. 3C).

**Table 2:** Median effective dose (ED_50_) for SE termination of intravenous anesthetics.

Experimental group	Number of animals (Analyzed/Censored)	ED_50_ (95 % CI)
SE + Mid	25/1	18 mg/kg (NC)
SE + Pro	22/2	20 mg/kg (NC)
SE + Dex	21/1	60 μg/kg (54.83 to 65.17)
SE + Esk	21/6	60 mg/kg (NC)

ED_50_ was determined using a dose-to-event survival analysis. Censored animals were those that died from GA-related causes before SE termination. NC, Not calculable; CI, confidence interval. The 95 % CI for these agents could not be reliably computed because the survival function did not fall below 50 % with sufficient data, or due to a high rate of censoring.

**Table 3:** Analysis of the initial dose in relation to the ED_50._

Experimental group	Initial dose	ED_50_	Relative initial intensity (%)
SE + Mid	18 mg/kg	18 mg/kg	100
SE + Pro	20 mg/kg	20 mg/kg	100
SE + Dex	60 μg/kg	60 μg/kg	100
SE + Esk	40 mg/kg	60 mg/kg	66.7

The relative initial intensity is calculated as: (initial dose / ED50) x 100 %.

### Safety of GAs on SE termination

**Fig. 4:**
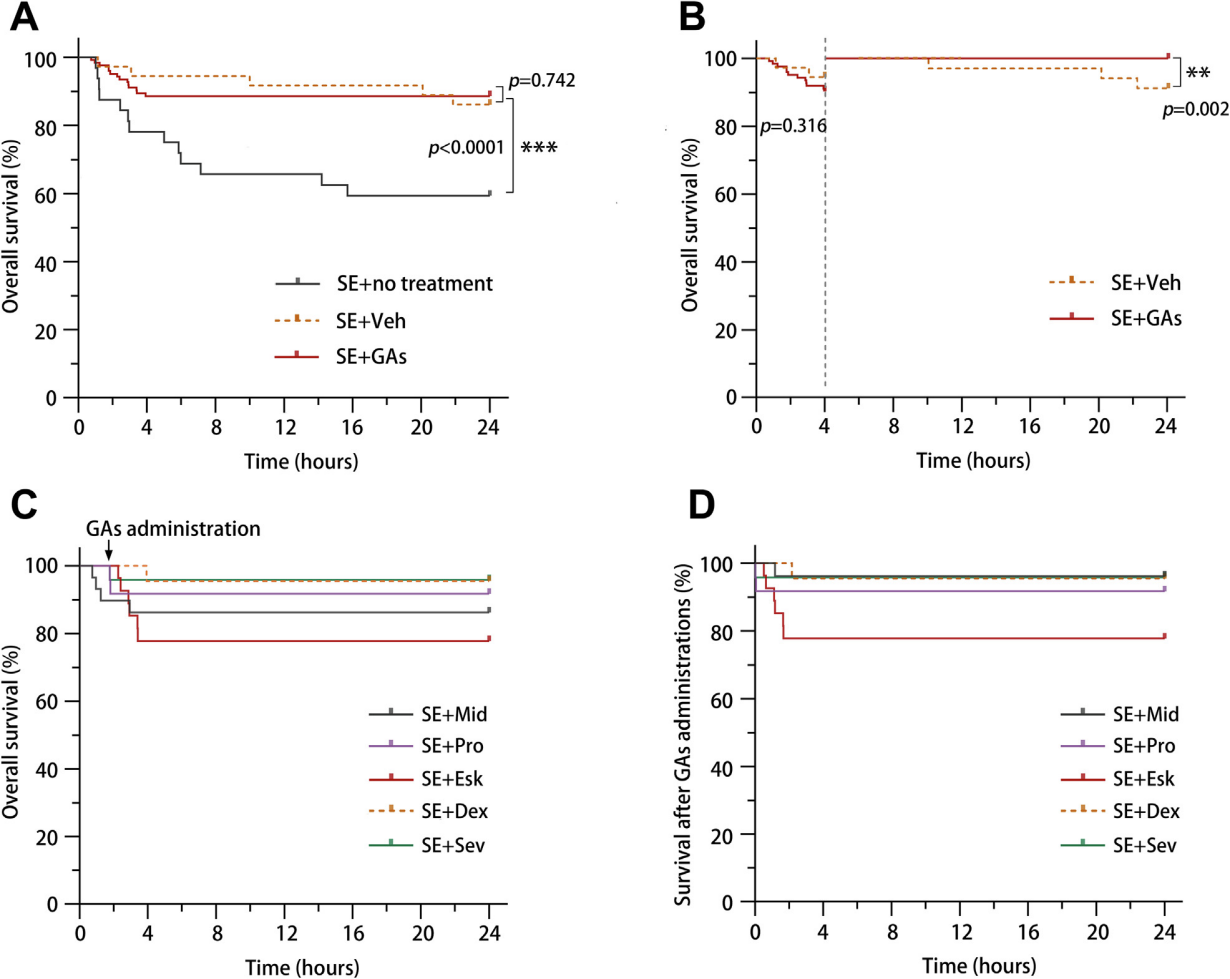
Survival of rats in different treatment groups. (A) Survival of rats in the SE + no treatment, SE + Veh, and SE + GAs groups within 24-hours of SE. (B) Landmark analysis discriminating between survivals within and after 4 h of SE onset. (C) Overall survival of rats treated with different GAs. (D) GA-related survival of rats treated with different GAs. Ctrl, control; Veh, vehicle; Mid, midazolam; Pro, propofol; Dex, dexmedetomidine; Esk, esketamine; Sev, sevoflurane. ^**^p < 0.01 and ^***^p < 0.0001. Data were statistically analyzed by the Log-rank test with Bonferroni’s correction, with significance for pairwise comparisons defined as **p < 0.0167.

The 24-hour mortality after SE in the untreated rats was 40.63 %. Mortality was significantly lower in the SE + GAs groups, whereas the reduction in the SE + Veh group was not statistically significant (Table 4). Moreover, there was no significant difference in overall survival between the SE + Veh and SE + GAs groups within 24 h of SE onset (Table 4 and Fig. 4A), leaving the specific contribution of GAs unclear.

**Table 4:** The overall 24-hour mortality.

Experimental group	Number of animals	Mortality within 24 h	
		Number of animals	Mortality (%)
Ctrl + no treatment	33	0	0
SE + no treatment	32	13	40.63**
Ctrl + Veh	36	0	0
SE + Veh	36	5	13.89
Ctrl + GAs	88	2	2.27
SE + GAs	123	14	11.38^##^

Ctrl, control; Veh, vehicle. ^**^p < 0.0033 compared with Ctrl + no treatment group; ^##^*p* < 0.0033 compared with SE + no treatment group. Data are analyzed by the Fisher’s exact test, with a *p* < 0.0033 considered significant after Bonferroni’s correction.

To isolate the effect of GAs, we performed a Landmark analysis of survival within and after 4 h. The 4 h was selected as the cutoff because RSE was completely controlled by GAs at this time point. The survival in the SE + GAs group was significantly higher than in the SE + Veh group after 4 h of SE onset, whereas there was no difference in survival within 4 h (Fig. 4B). Since three rats in the SE + Mid group died before midazolam injection (Fig. 4C), GAs-related mortality was recalculated from the beginning of GAs injection (Fig. 4D). Although treatment with esketamine resulted in the highest number of deaths, there was no statistical difference in survival between the five groups (Fig. 4D). Sex did not affect the overall mortality (Supplementary Fig. 2C). One control rat treated with propofol (Ctrl + Pro group) and one treated with esketamine (Ctrl + Esk group) died because of respiratory arrest.

Results of the blood gas analysis are listed in Supplementary Table 5. All rats in the SE + Veh or SE + GAs groups developed acidosis, with those in the SE + Veh group showing predominantly metabolic acidosis, while the GAs-treated rats exhibited both respiratory and metabolic acidosis. However, no differences were detected in arterial blood gas parameters between the groups.

### Effect of GAs on neurological outcomes

**Fig. 5:**
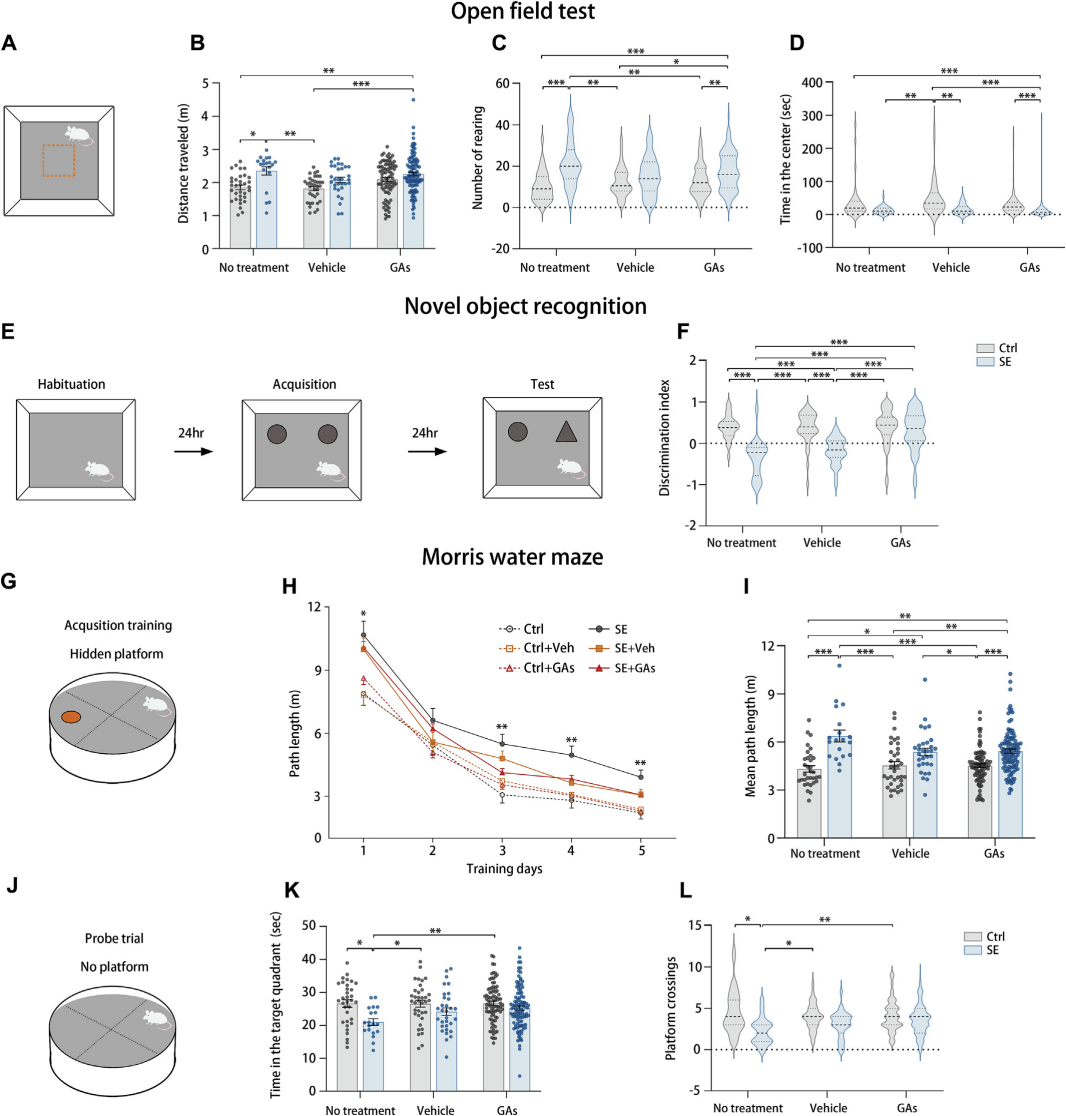
The effect of GAs on neurological function following SE. (A) Schematic diagram of the open field test. (B) Total distance traveled, (C) number of rearing, and (D) time spent in the center over 5 min on Day 5 following SE. (E) Schematic diagram of the novel object recognition test. (F) The discrimination index in the test session 24 h after the acquisition session on Day 7 following SE. (G) Schematic diagram of the acquisition training in the hidden platform of Morris water maze. (H) Mean path length across the 5 consecutive days of the acquisition training from Day 7 to 11 following SE. (I) Mean path lengths across all trainings. (J) Schematic diagram of the probe trial. (K) Time in the target quadrant and (L) number of platform crossing on Day 12 following SE. Ctrl, control; Veh, vehicle. Ctrl + no treatment, n = 33; SE + no treatment, n = 19; Ctrl + Veh, n = 36; SE + Veh, n = 31; Ctrl + GAs, n = 86; SE + GAs, n = 109. Error bars represent the standard error of the mean (SEM). *p < 0.05, ^**^p < 0.01, and ^***^p < 0.0001. Data were statistically analyzed by a two-way ANOVA or Scheirer-Ray-Hare test followed by a Sidak’s test in the open field and novel object recognition tests and by a two-way repeated ANOVA followed by a Sidak’s test in the Morris water maze test. Distance traveled: two-way-ANOVA_sex x group_*F*_(5,302)_ = 0.66, *p* = 0.658; two-way-ANOVA_group_*F*_(5,302)_ = 6.61, *p* < 0.0001. Rearing: two-way-ANOVA_sex x group_*F*_(5,302)_ = 0.83, *p* = 0.531; two-way-ANOVA_group_*F*_(5,302_ = 7.34, *p* < 0.0001. Time in the center: two-way-ANOVA_sex x group_*F*_(5,302)_ = 0.93, *p* = 0.463; two-way-ANOVA_group_*F*_(5,302)_ = 8.01, *p* < 0.0001. Discrimination index: two-way-ANOVA_sex x group_*F*_(5,302)_ = 0.16, *p* = 0.976; two-way-ANOVA_group_*F*_(5,302)_ = 16.92, *p* < 0.0001. Path length: two-way-repeated-ANOVA_sex x group_*F*_(5,302)_ = 1.38, *p* = 0.232; two-way-repeated-ANOVA_group_*F*_(5,302)_ = 12.76, *p* < 0.0001; two-way-repeated-ANOVA_sex_*F*_(1,1302)_ = 2.46, *p* = 0.118. Mean path length: two-way-ANOVA_sex x group_*F*_(5,302)_ = 1.39, *p* = 0.227; two-way-ANOVA_group_*F*_(5,302)_ = 12.83, *p* < 0.0001. Time in the target quadrant: two-way-ANOVA_sex x group_*F*_(5,302)_ = 0.90, *p* = 0.483; two-way-ANOVA_group_*F*_(5,302)_ = 3.39, *p* = 0.005. Platform crossing: two-way-ANOVA_sex x group_*F*_(5,302)_ = 0.33, *p* = 0.897; two-way-ANOVA_group_*F*_(5,302)_ = 4.13, *p* = 0.001.

In the OF test, all rats with SE showed hyperactivity, as evidenced by a greater total distance traveled (horizontally) and a higher degree of rearing (vertically) compared to controls (Fig. 5B and C). Rats that experienced SE spent less time exploring the center of the open field, indicating a higher anxiety response. Treatment with ASMs or GAs did not alter this anxiety-like behavior (Fig. 5D). In the absence of SE, ASMs or GAs alone did not cause changes in spontaneous behavior.

Next, cognition including episodic memory and spatial navigation, were tested using the ORM and MWM tests, respectively [28]. In the ORM test, rats in the SE + no treatment group showed no preference for the novel object, as reflected in the negative DI, indicating a deficit of episodic memory. Similarly, rats in the SE + Veh group were unable to discriminate the novel object from the familiar one. In contrast, treatment with GAs significantly reversed this impaired episodic memory (Fig. 5F) without affecting the predisposition to explore objects (Supplementary Fig. 1A and B). Control animals without SE had an intact episodic memory despite being exposed to ASMs or GAs.

In the hidden platform MWM, path length instead of escape latency was used to evaluate the ability of spatial navigation, because rats with SE swam faster than controls (Supplementary Fig. 1C). Rats in the SE + no treatment group required a longer distance traveled to reach the platform (Fig. 5I), especially on Days 1, 3, 4, and 5 (Fig. 5H), suggesting impaired spatial learning. SE rats exposed to ASMs or GAs also learned the task more slowly than corresponding controls. There was a slight reduction in mean path length in SE + Veh and SE + GAs relative to the SE + no treatment group, but it did not reach a significant level. Spatial memory deficits were evident in SE rats with no treatment, as showed by less time spent searching in the target quadrant and less platform crossing (Fig. 5K and L). There was a trend for rats in the SE + GAs group to exhibit improved spatial memory compared with SE rats without treatment (Time in the target quadrant: *p* = 0.063; Platform crossing: *p* = 0.064). There were no differences in all data between the control groups. Moreover, rats with SE were unaffected in the visible platform version of the water maze, indicating that their visual, motor, and motivational systems remained intact (Supplementary Fig. 1E). Sex did not affect all the results of the behavioral tests (Supplementary Fig. 2D-J).

## Comparison between different GAs for neurological outcomes

**Fig. 6:**
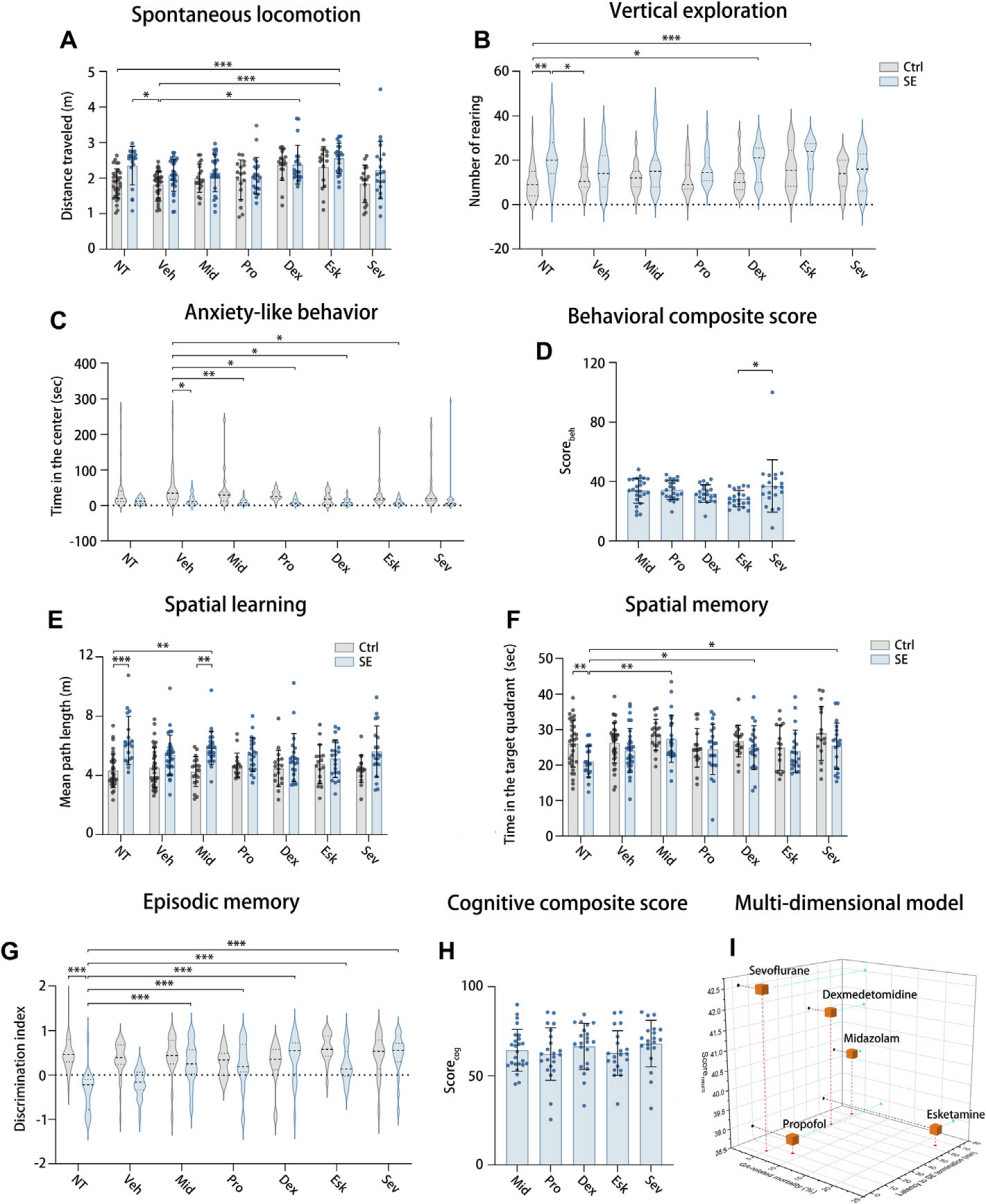
Comparison between different GAs on neurological outcomes. (A) Spontaneous locomotion as measured by the total distance traveled, (B) vertical exploration quantified as the number of rearing, and (C) time spent in the center over 5 min, reflecting anxiety-like behavior in the open field test on Day 5 following SE. (D) The composite score of behavior. (E) Spatial learning, represented by the mean path length before achieving the platform for 5 consecutive days of the acquisition training from Day 7 to 11 following SE. (F) Spatial memory, evaluated as the time in the target quadrant on Day 12 following SE. (G) Episode memory, indicated by the discrimination index in the test session on Day 7 following SE. (H) The composite score of cognition. (I) The model of a three-dimensional diagram. The orange squares represent the spatial distribution of 5 different GAs. The dots and corresponding dashed lines represent the coordinate and projected lines of each drug. Ctrl, control. Ctrl + no treatment, n = 33; SE + no treatment, n = 19; Ctrl + Vehicle, n = 36; SE + Vehicle, n = 31; Ctrl + Midazolam, n = 19; Ctrl + Propofol, n = 17; Ctrl + Dexmedetomidine, n = 18; Ctrl + Esketamine, n = 16; Ctrl + Sevoflurane, n = 16; SE + Midazolam, n = 25; SE + Propofol, n = 22; SE + Sevoflurane, n = 20; SE + Dexmedetomidine, n = 21; SE + Esketamine, n = 21. Error bars represent the standard error of the mean (SEM). *p < 0.05, ^**^p < 0.01, and ^***^p < 0.0001. Data were statistically analyzed by a two-way ANOVA or Scheirer-Ray-Hare test followed by a Sidak’s test in the open field, novel object recognition, Morris water maze tests, and composite score. Distance traveled: two-way-ANOVA_sex x group_*F*_(13,286)_ = 1.16, *p* = 0.307; two-way-ANOVA_group_*F*_(13,286)_ = 4.76, *p* < 0.0001; two-way-ANOVA_sex_*F*_(1,286)_ = 3.82, *p* = 0.052. Rearing: two-way-ANOVA_sex x group_*F*_(13,286)_ = 1.01, *p* = 0.440; two-way-ANOVA_group_*F*_(13,286)_ = 3.67, *p* < 0.0001; two-way-ANOVA_sex_*F*_(1,286)_ = 0.74, *p* = 0.389. Time in the center: two-way-ANOVA_sex x group_*F*_(13,286)_ = 0.82, *p* = 0.643; two-way-ANOVA_group_*F*_(13,286)_ = 3.37, *p* < 0.0001; two-way-ANOVA_sex_*F*_(1,286)_ = 0.41, *p* = 0.524. Discrimination index: two-way-ANOVA_sex x group_*F*_(13,286)_ = 1.21, *p* = 0.275; two-way-ANOVA_group_*F*_(13,286)_ = 7.44, *p* < 0.0001; two-way-ANOVA_sex_*F*_(1,286)_ = 0.73, *p* = 0.395. Mean path length: ANOVA_sex x group_*F*_(13,286)_ = 1.54, *p* = 0.104; two-way-ANOVA_group_*F*_(13,286)_ = 5.21, *p* < 0.0001; two-way-ANOVA_sex_*F*_(1,286)_ = 1.78, *p* = 0.183. Time in the target quadrant: two-way-ANOVA_sex x group_*F*_(13,286)_ = 0.83, *p* = 0.627; two-way-ANOVA_group_*F*_(13,286)_ = 2.02, *p* = 0.019; two-way-ANOVA_sex_*F*_(1,286)_ = 0.73, *p* = 0.393. Platform crossing: two-way-ANOVA_sex x group_*F*_(13,286)_ = 0.70, *p* = 0.759; two-way-ANOVA_group_*F*_(13,286)_ = 2.35, *p* = 0.006; two-way-ANOVA_sex_*F*_(1,286)_ = 0.003, *p* = 0.976. Score_beh_: two-way-ANOVA_sex x group_*F*_(4,99)_ = 2.47, *p* = 0.051; two-way-ANOVA_group_*F*_(4,99)_ = 2.55, *p* = 0.044; two-way-ANOVA_sex_*F*_(1,99)_ = 1.18, *p* = 0.279. Score_cog_: two-way-ANOVA_sex x group_*F*_(4,99)_ = 0.80, *p* = 0.527; two-way-ANOVA_group_*F*_(4,99)_ = 0.86, *p* = 0.491; two-way-ANOVA_sex_*F*_(1,99)_ = 0.04, *p* = 0.834.

In the OF test, rats treated with dexmedetomidine or esketamine performed significant hyperactive behavior, while rats exposed to the other three GAs exhibited behaviors similar to their corresponding controls (Fig. 6A and B). Anxiety-like behavior was observed in all SE groups treated with GAs except for sevoflurane (Fig. 6C). Thus, using a composite score that takes into account outcomes of multiple behavioral tests, treatment with sevoflurane had the least impact on behavior, while exposure to esketamine had the most (Fig. 6D). In the tests of cognition, SE rats treated with midazolam showed impaired spatial learning but intact spatial memory (Fig. 6E and F). Dexmedetomidine and sevoflurane rescued the impaired spatial memory induced by SE (Fig. 6F). All five GAs had the same protective effect on episodic memory deficit after SE (Fig. 6G). The composite score of cognition was also calculated, and no significant difference was found between the five groups (Fig. 6H). Finally, the overall composite score of neurological outcomes was calculated by adding the scores of behavior and cognition.

### A proposed multi-dimensional model of GA selection for RSE

To comprehensively evaluate the role of different GAs for treating SE in terms of three aspects: efficacy, safety, and effect on neurological outcomes, we plotted a three-dimensional diagram with GAs-related mortality, latency to SE termination, and the composite score of neurological outcomes on the x, y, and z axes, respectively. This model suggests that the optimal choice of GAs was sevoflurane, whereas the worst choice was esketamine (Fig. 6I).

## Discussion

In this study, we found that treatment with GAs effectively stopped RSE and reduced 24-hour mortality in a time-phased manner. Furthermore, GAs treatment reduced short-term cognitive deficits after SE, especially episodic memory. Last, sevoflurane might be the optimal GA for RSE treatment based on our proposed multi-dimensional model.

Consistent with previous studies [29], immature rats developed generalized seizures accompanied by continuous rhythmic epileptiform activity on EEG for 8 to 10 h. In clinical SE, the efficacy of BZDs decreases dramatically as SE duration increases [13]. Similarly, when diazepam was administered 45 min after SE onset in this study, it did not terminate SE in any rat. On this basis, we sequentially administered the second-line ASM, phenytoin and found that 99.4 % of the rats still experienced ongoing SE, indicating pharmacoresistance to phenytoin. In this situation, this preclinical model closely resembled pediatric RSE in the clinical setting.

GAs are commonly used as the third-line therapy for RSE, but there is a lack of robust evidence to guide the optimal selection [4,5]. GAs have different pharmacological properties, which may be associated with distinct benefits and risks. Among the five agents tested in this study, midazolam, propofol, and esketamine are commonly used for treating RSE [5,6,30], while the use of sevoflurane or dexmedetomidine has been reported in case reports [24,31,32].

In this study, propofol and sevoflurane were the most effective drugs in terminating RSE. Both drugs had a fast onset of action with relatively low doses, consistent with previous studies [24,33]. Their efficacy may be related to their pharmacological effects in widespread brain regions and via multiple drug-binding sites [34,35]. N-methyl-D-aspartate (NMDA), γ-aminobutyric acid type A (GABA_A_), α-amino-3-hydroxy-5-methyl-4-isoxazolepropionic acid (AMPA), glycine, serotonin, and nicotinic acetylcholine receptors and potassium, sodium, and calcium channels are sensitive to these two GAs at clinically relevant concentrations. This multi-target action provides a broader anticonvulsant mechanism, which simultaneously enhances inhibitory neurotransmission via GABA_A_ and glycine receptors and suppresses excitatory signaling via NMDA and AMPA receptors, thereby directly counteracting the network hyperexcitability that sustains SE. Furthermore, the BDZs-sensitive γ2-subunit of the GABA_A_ receptor is internalized with increasing duration of SE, resulting in progressive loss of BDZs efficacy [36,37]. In comparison, propofol and sevoflurane can bind to other functional populations of GABA_A_ receptors, such as α- and β-subunits, to control seizure by enhancing GABA-induced chloride current [34,35]. Crucially, this allows them to bypass the GABA_A_ receptors refractoriness that limits midazolam. This might explain the higher efficacy of propofol and sevoflurane compared to midazolam.

Dexmedetomidine is a central highly selective α-2 adrenergic agonist frequently used for anesthesia in the OR and sedation in the ICU [38]. The effect of dexmedetomidine on seizures remains unclear, although it may be an option for treating SE based on a few animal studies and case reports [31,32,39]. Here, the use of dexmedetomidine could stop sustained SE, but required a relatively long time. An explanation may be that dexmedetomidine modulates seizure activity by affecting the adrenergic system via two different mechanisms. In the early phase, dexmedetomidine acts dominantly on the presynaptic α-2 adrenoreceptor, thereby reducing noradrenaline release through a negative feedback system. Therefore, dexmedetomidine treatment counteracts the increased release of endogenous norepinephrine induced by sustained SE. However, norepinephrine is a potent anticonvulsant that affects seizure initiation, spread, and termination [40]. In contrast, dexmedetomidine preferentially binds to postsynaptic α-2 adrenoreceptor at a later stage, leading to inhibition of neuronal excitability and regulation of the seizure threshold [41], ultimately terminating SE.

Altered receptor trafficking, including a decrease in GABA_A_ receptors and an increase in NMDA receptors, is one of the important pathophysiological changes in the late stage of SE[5]. This theory provides a strong rationale for the use of ketamine (a noncompetitive NMDA receptor antagonist) in RSE. Increasing pediatric and adult data suggest the efficacy of ketamine in treating RSE and super RSE [42,43], which prompted us to investigate the effectiveness of esketamine, the S-enantiomer of ketamine, in the experimental model of RSE. Compared with ketamine (a racemic mixture of different R- and S-enantiomers), esketamine has a higher affinity for NMDA receptors [44], thus fewer side effects and quicker clearance are evident due to the lower dose necessary to produce an equianesthetic effect. However, we found that high doses of esketamine were needed to control SE, and more interestingly, esketamine rapidly stopped behavioral SE but took longer (63.86 min) to terminate electrographic SE. Our findings are in line with previous animal and clinical studies that ketamine is ineffective in the too-early phase (animals: within 1 h of SE onset; humans: hours to some days after SE onset) [45,46], indicating that the occurrence of NMDA receptor trafficking may be delayed. Another important finding was the inconsistency in latency to termination between electrographic SE and behavioral SE, highlighting the importance of continuous EEG monitoring during SE treatment. It has been reported that the clinical efficacy of ketamine is associated with a heterogeneous EEG pattern. In addition to burst suppression (BS), diffuse slowing and β activity should be considered the targets to achieve [47]. Accordingly, different markers of electrographic SE termination may be required to evaluate the efficacy of different GAs more accurately.

The dosing regimen of IAs in our study closely mimicked the clinical paradigm of an initial induction dose followed by titration, with EEG monitoring used to precisely define the endpoint of SE termination. To control for the confounding influence of this dosing regimen on the latency to SE termination, we determined the ED_50_ for each IA and calculated the “relative initial intensity”, defined as the ratio of the initial dose to the ED_50_. We found that the initial dose matched the ED_50_ exactly for midazolam, propofol, and dexmedetomidine, whereas for esketamine it covered only 66.7 % of its ED_50_, partly explaining the longer latency in the SE + Esk group. However, our pilot studies found that an initial esketamine dose equivalent to its ED_50_ (60 mg/kg) induced severe respiratory depression or even death in approximately 70 % of rats, indicating an exceedingly narrow therapeutic window that precludes such dosing without mechanical ventilation. For the VA sevoflurane, the dosing regimen followed its pharmacodynamic properties and clinical induction standards. We used a high initial concentration of 4 %, approximately twice the minimum alveolar concentration (MAC), the standard measure of VA potency required to prevent movement in 50 % of subjects responding to a noxious stimulus. This induction dose rapidly reduced epileptiform amplitude within 2 min and produced an isoelectric EEG withing 5 min, demonstrating its sufficiency for rapid SE termination. The concentration was then lowered to a maintenance level of 1.5–2 %, about 1 MAC, for 5 h to maintain a seizure-free state.

We found that treatment with GAs significantly improved survival. These results contrast with previous clinical observation, which demonstrated that the use of GAs in SE was associated with higher mortality and increased infection (7–9). The discrepant results may be attributed to several factors. First, the previous clinical studies were observational in design, which is prone to selection bias. In comparison, our work was a randomized controlled animal study, and the equal SE severity in GAs and non-GAs treatments was confirmed quantitatively by the Racine scale and EEG spectral power analysis. Second, patients who receive continuous infusion of GAs are necessarily intubated and mechanically ventilated. Prolonged mechanical ventilation increases the risk of respiratory tract infection, diaphragmatic weakness, prolonged hospital stay, and mortality [48]. To control these two confounders (intubation and mechanical ventilation), we conducted non-intubated anesthesia with preserved spontaneous breathing. Under this paradigm, when SE was completely controlled by GAs (4 h after SE onset), there was no death within the following 20 h, whereas three rats treated with vehicles died because of persistent SE, indicating that GAs treatment for several hours without mechanical ventilation improved survival by effectively controlling SE. Third, GAs were administered to maintain the seizure-free status for 5 h, which was much shorter than the duration of continuous infusion of GAs in the clinical setting. Therefore, common life-threatening complications associated with prolonged continuous infusions of GAs, such as propofol infusion syndromes, severe cardiovascular suppression, and paralytic ileus were avoided in our study.

Regarding the safety of the different GAs, there was no difference in GA-related mortality between the five anesthetic agents. However, mortality after esketamine treatment was relatively higher due to the unusually large dose, which suggested that the safety of esketamine in SE treatment without mechanical ventilation was lower than the other GAs. Conversely, ketamine is gaining popularity for the treatment of RSE owing to its favorable safety profile [42]. Theoretically, due to its unique pharmacological properties, ketamine is less likely to cause cardiorespiratory compromise than other GAs, making it a promising treatment that avoids endotracheal intubation [42]. However, these data are limited to isolated case reports and retrospective studies [43,46,49]. In this study, post-anesthesia blood gas analysis revealed metabolic acidosis in the SE + Veh group, which aligns with the expected lactic acidosis from persistent convulsions and resultant hypoperfusion [50]. Conversely, rats in all GAs-treated groups exhibited metabolic acidosis even after SE termination, pointing to a potential distinct mechanism. A plausible cause is GA-induced persistent hypotension during the prolonged maintenance phase, attributable to the known hemodynamic suppression at the mean doses required to terminate SE (Supplementary Table 4). More importantly, the occurrence of significant carbon dioxide accumulation and respiratory acidosis in almost all SE + GAs groups after 5 h of anesthesia suggests that prolonged continuous use of GAs without mechanical ventilation is not a safe strategy.

Another key finding was that treatment with GAs did not affect the hyperactive or anxiety-like behavior caused by SE but significantly improved cognition, especially episodic memory. These neuroprotective effects are similar to the previous studies [27,51], but some differences remain. Previous studies used single-sex (male) adult rats and focused on the effects of polytherapy (dual or triple) compared with monotherapy in SE on long-term spatial memory [27,51]. The GAs involved in these studies were only midazolam and ketamine. In our study, both male and female PND 21 rats were used, corresponding to childhood in humans [52]. We and other researchers previously showed that early exposure to GAs might cause neurotoxicity in the developing brain and cognitive dysfunction. In this study, we did not observe any negative influence of GAs on short-term behavior and cognition, either with or without SE. The reason for this inconsistency might be the different maturity of the developing brain when animals are exposed to GAs. The immature brain is susceptible to GAs-induced damage at the peak of synaptogenesis [53] which ranges from the trimester of *in-utero* to the first two years after birth in humans and from postnatal day 7 to day 10 in rodents [54]. In this study, however, the more mature brain is less sensitive to GAs-induced neurotoxic insult. In addition, the improvement in episodic memory deficit by GAs without concurrent anxiolytic effects provides robust evidence that the cognitive benefit is a direct primary effect rather than secondary to emotional changes. To control for the confounding effects of anxiety on cognitive behavior, we also assessed spatial learning and memory using the MWM, a paradigm less influenced by anxiety [55,56]. Since we did not monitor seizure activity after SE recovery, we could not definitively exclude an influence of spontaneous recurrent seizures (SRSs) on behavior and cognition, however, we did not observe any behavioral seizures in the rats during behavioral testing. In fact, epileptogenesis is age-dependent in experimental models [57]. Roch et al reported that the incidence of SRSs after lithium-pilocarpine-induced SE is 28.57 % in PND21 rats [58], significantly lower than the nearly 100 % prevalence in adult rats [59]. Besides, the latency to SRSs onset in immature rats was also significantly prolonged, with a mean of 70 days [58] compared to 15 days in adult rats [59]. Therefore, our behavioral tests were completed within 12 days post-SE, thereby minimizing potential confounding effects.

Hyperactivity is a long-term behavioral sequela of SE across multiple animal models [60,61]. Our findings in the short term after lithium-pilocarpine-induced SE confirm this and further demonstrate that the choice of GAs critically modulates its severity. Specifically, a trend of improvement with first- and second-line ASMs was offset by the potent hyperactive effects of dexmedetomidine and, most strikingly, esketamine. This drug-specific effect was summarized by the composite behavioral score, which ranked esketamine lowest and sevoflurane highest. The pronounced hyperactivity from esketamine is consistent with its action as an NMDA receptor antagonist, potentially involving dopamine release in the thalamic motor nuclei [62]. In contrast, cognitive composite scores were similar across GA groups, with a trend towards greater efficacy in rescuing episodic versus spatial memory, suggesting additional protection beyond the hippocampus, possibly in the perirhinal cortex [63]. Moreover, the dissociation between the rescued episodic memory and the unchanged spatial memory at this early post-SE stage highlights a key boundary of GAs-mediated neuroprotection. This discrepancy is likely attributable to the timing of assessment, as a longer post-SE interval is typically required to detect robust spatial memory deficits and their rescue effect [27].

To find the optimal GA for RSE treatment, we plotted a 3-dimensional diagram in which the three axes represented safety, efficacy, and effect on neurological prognosis. This analysis suggests that sevoflurane was the best choice, while esketamine was the worst. VAs, such as isoflurane and desflurane, are used in treating RSE and are effective in inducing BS [64]. Sevoflurane was seldom used for RSE because of its potential pro-epileptogenic effects [65]. However, this effect is manifested by inducing epileptiform activity on the EEG, yet rarely inducing seizures [65]. Those non-convulsive epileptiform discharges are thought to be a variation of the BS pattern commonly observed in most subjects with a high dose of sevoflurane [65]. We also observed epileptiform activity during anesthesia induction of sevoflurane, which differed from the high-frequency epileptiform discharges of SE in that it alternated with isoelectric potentials, and we, therefore, recognized it as a BS pattern (Fig. 3C). VAs have many advantages in the treatment of RSE. In particular, as short-acting drugs, they are easy to titrate to achieve the BS pattern, and drug clearance happens almost exclusively by pulmonary exhalation, thus reducing the risk of severe side effects from prolonged exposure [66]. Hypotension is a common side effect of VAs, similar to the IAs used in SE [64]. Last, VAs are unlikely to cause the early onset of tolerance and ceiling effects, which are characteristic of IAs [66]. However, there are significant limitations of VAs for SE. First, prolonged use of VAs leads to elevated levels of inorganic serum fluoride, which may cause hepatic injury. Second, fluorinated byproducts of sevoflurane have the potential to bind with carbon dioxide to form Compound A, which may impair renal function. Third, presumed or proven malignant hyperthermia is a contraindication to VAs therapy [67]. Furthermore, the use of VAs outside the OR requires specific VA delivery systems and anesthetic exhaust scavenging systems, which are also limitations for their use.

This study has several limitations. First, the experimental model of SE was developed by chemical administration, which does not recapitulate the full spectrum of human SE features. For example, the etiology of pediatric SE is complex and diverse. The etiology may affect the severity of SE, mortality, responses to treatment, and neurological prognosis [5,68]. Febrile SE is the most common cause of pediatric SE overall, accounting for approximately one-third of cases [69]. Although the hyperthermic seizure model has been developed, its use is also limited because hyperthermic seizures are triggered by exogenous forceful heating of the body rather than by endogenous pyrogenic substances observed in children [70]. Moreover, febrile seizures only last for approximately 20 min, and behavioral seizures rarely progress to generalized tonic seizures [71]. Hence, this model is not suitable for our study. Second, our study focused on assessing neurological prognosis during the early phase (5–12 days) after RSE. However, we did not evaluate long-term alterations in behavior and cognition, nor did we observe the epileptogenesis, which is also a critical sequela of RSE. This design was adopted to serve our primary objective: to isolate the potential direct neurotoxic effects of GAs on the developing brain from the confounding influence of subsequent occurring SRSs. Future studies will be essential to evaluate these long-term sequelae. Additionally, all behavioral tests were conducted during the light phase of the circadian cycle. While this may influence the animals’ natural activity patterns and responses, this schedule was maintained for practical feasibility. To mitigate this potential confound and reduce testing-related stress, we standardized the testing environment with dim illumination (3 lx), a widely accepted method that increases the sensitivity of behavioral assays even during the less active phase [72]. Future investigations performing tests during the dark phase could provide a complementary perspective on neurological outcomes. Third, the absence of neuropathological examination limits the interpretation of our findings at a structural level. Although GAs conferred clear cognitive protection, we cannot ascertain whether this functional benefit correlates with the mitigation of specific structural pathologies, such as neuronal loss, synaptic reorganization, or gliosis, which are hallmarks of RSE. A key future direction will be to combine behavioral assessment with histology to bridge this gap.

## Conclusion

In conclusion, our preclinical findings provide supportive evidence that the benefits of GAs outweigh the risks of treating clinical RSE. Furthermore, from a balanced consideration of efficacy, safety, and neurological sequelae, our data suggest that VAs may be the optimal GA for treating RSE and warrant further clinical evaluation.

## Compliance with ethics requirements

All Institutional and National Guidelines for the care and use of animals (fisheries) were followed.

## Declaration of competing interest

The authors declare that they have no known competing financial interests or personal relationships that could have appeared to influence the work reported in this paper.
