# Supplementary material for: Effects of general anesthetics on seizure termination, mortality, and neurological prognosis in a rat model of pediatric refractory status epilepticus: A comparative randomized controlled study
Source: J Adv Res. 2025 Nov 2;86:527–41. doi: 10.1016/j.jare.2025.10.076 (PMC13453627; doi:10.1016/j.jare.2025.10.076)
Supplement: Supplementary file 1 [file mmc1.docx]

**Supplementary Materials**

Title: Effects of general anesthetics on seizure termination, mortality, and neurological prognosis in a rat model of pediatric refractory status epilepticus: a comparative randomized controlled study

Journal: *Journal of Advanced Research*

Authors: Bo Chen, Yuping Zhao, Na Shen, Feng Liu, Li Jiang, Yuanlin Ma, Xin Tian, Patrick Kwan.

**Correspondence to: Patrick Kwan**, FRACP, PhD., Department of Neurology, the First Affiliated Hospital of Chongqing Medical University, Chongqing Key Laboratory of Neurology, Chongqing 400016, P.R. China.

E-mail: [patrick.kwan@monash.edu](mailto:liuhl75@163.com)

**This PDF file includes:**

Supplementary Methods

Supplementary Tables 1 to 5

Supplementary Figures 1 and 2

References for supplementary materials reference citations

**Supplementary Methods**

**Electrodes implantation and EEG recordings**

Rats (PND 16) were fixed in a stereotaxic apparatus (RWD Life Science Co., Ltd., Shenzhen, China). Two stainless steel screws with attached wire leads (Pinnacle Technology, Inc., KS, USA) were inserted into the skull above the bilateral parietal cortex (AP: -3 mm, ML: ±2 mm) as recording electrodes, a location established for effectively capturing the widespread cortical epileptiform activity in the lithium-pilocarpine-induced status epilepticus model ^1, 2^. One ground screw was positioned above the prefrontal cortex (AP: +1 mm, ML: +2 mm) and a reference screw above the cerebellar cortex (AP: -12 mm, ML: -2 mm), a site chosen for its electrical stability and distance from the primary regions of seizure activity ^2^. All electrode leads were connected to a prefabricated headmount (Pinnacle Technology) and sealed with dental acrylic.

Each rat (PND 21) was placed in a separate recording chamber with its headmount connected to a preamplifier. All amplified signals were transmitted to the conditioning/acquisition system via a tether cable and a low-torque commutator (Pinnacle Technology). The EEG signals were digitized at 400 Hz and band-pass filtered at 40-100 Hz. Signal acquisition was performed using the Sirena acquisition suite (Pinnacle Technology). Baseline EEG was recorded for 30 min before pilocarpine injection, and EEG monitoring continued for 24 hours.

The PSD analysis was performed using Welch's method in MATLAB (MathWorks, MA, USA) described previously with minor modifications^2^. Briefly, raw EEG recordings were transformed into EDF format. The signal from the left parietal channel was selected for analysis because signals of both channels showed a similar pattern during SE, and the signals were filtered using a Butterworth filter (bandpass: 0.1-125 Hz; notch filter at 50 Hz). A 60-second window was selected to obtain a satisfied spectral and temporal resolution. The PSD (μV^2^/Hz; 0.1-125 Hz) at 1 min before treatment with GAs or vehicles was derived by integrating the power spectra obtained via the fast Fourier transform over 60-second epochs with a 50% overlap.

**Behavioral tests**

The abnormal behavior after SE was evaluated by the OF test. Rats were placed in the center of a chamber (100×100×50 cm) and allowed to explore the arena for 5 minutes (Fig. 5A). Spontaneous activity was recorded by a video tracking system, the total distance traveled and time spent in the center area, and numbers of rearing were measured automatically by OF motion-detection software (Zhenghua Biotech Co., Ltd., Hebei, China).

The episodic memory after SE was evaluated using the ORM test. The ORM test comprised two sessions (Fig. 5E). Prior to the first session, rats were handed for 5 minutes, followed by habituation to the experimental apparatus (50×50×40 cm) with 10 minutes per day for two consecutive days. In the acquisition session, rats were placed in the apparatus containing two identical objects and allowed to explore freely for 5 minutes. After a 24-hour delay, one of the objects was replaced with a novel one, and rats were allowed 3 minutes of exploration (test session). All behaviors in the acquisition and test sessions were video recorded and analyzed by a researcher blinded to the experimental design. Exploration times were calculated as a discrimination index (DI, DI = (T_novel_ - T_familiar_)/(T_novel_ + T_familiar_)).

Navigation and spatial memory after SE were evaluated using the MWM test. For the hidden platform training, a platform was submerged in a black circular tank (150 cm in diameter) filled with water (22 ± 2 °C) with ink (Fig. 5G). Rats were trained four trials per day for consecutive 5 days, and the escape latency and swimming path in each trial were recorded. In each trial, rats were placed in the tank at a random starting position and allowed to search for the platform within 60 seconds. If rats failed to find the platform within 60 seconds, they were guided to the platform and allowed to stay on it for 10 seconds, and then the escape latency was recorded as 60 seconds. Twenty-four hours after the last training trial, rats were given a probe trial in which the original platform was removed (Fig. 5J). Rats were placed in the opposite quadrant to the platform quadrant and allowed to swim for 60 seconds. The number of platform crossings and time spent in the targeted quadrant were recorded. For the visible platform test, a white cover that extended 2 cm above the water level was placed on top of the platform, and two white flags were affixed to the sides of the platform, extending 10 cm above the water. The platform was moved to a novel quadrant in the tank for each trial, and animals were tested for four trials (Supplementary Fig. 1D). A video camera mounted directly above the tank was used to record all sessions for later analysis with tracking software (Zhenghua Biotech Co., Ltd.).

**Composite scores for outcomes of behavioral tests**

To generate the overall neurological composite score (Score_neuro_), the raw data of each behavioral test was first normalization transformed using the Min-Max method, which linearly scaled the data from 0 to 1, and 0 represented the most neurological deficits while 1 indicated no impairment. Score = (X-X_min_)/(X_max_-X_min_) or Score = (X_max_-X)/ (X_max_-X_min_), where X = the raw data of each test, X_min_ = minimal data of the test for the entire group, and X_max_ = maximal data of the test. Then, the scores of each behavioral test were summed and divided by the total number of tests to calculate the overall Score_neuro_ = Σscore/n; where n = total number of tests performed in the animals.

**Table 1. Drugs used in this study**

| **Drugs** | **Manufacturers** | **Cat. No.** | **Vehicle** |
| --- | --- | --- | --- |
| Lithium chloride | Macklin Biochemical | L812623 | Distilled water |
| Pilocarpine hydrochloride | Macklin Biochemical | P875258 | Distilled water |
| Scopolamine methyl bromide | Macklin Biochemical | S835305 | Distilled water |
| Phenytoin sodium | Macklin Biochemical | P993596 | 40% propylene glycol  10% ethanol  1.5% benzyl alcohol |
| Diazepam | Jinyao Pharmaceuticals Co., Ltd. | 1407061 | 5% glucose saline |
| Propofol | Fresenius Kabi | PA0566-021-002 | Intralipid |
| Midazolam | Enhua Pharmaceuticals Co., Ltd. | 20220721 | Normal saline |
| Dexmedetomidine | Hengrui Pharmaceuticals Co., Ltd. | 20090248 | Normal saline |
| Esketamine | Hengrui Pharmaceuticals Co., Ltd. | 20244192 | Normal saline |
| Sevoflurane | Maruishi Pharmaceutical Co., Ltd. | 20170389 | 90% O_2_ |

**Table 2. Sex distribution of animals in the main groups**

|  | Ctrl+no treatment | SE+no treatment | Ctrl+Veh | SE+Veh | Ctrl+GAs | SE+GAs |
| --- | --- | --- | --- | --- | --- | --- |
| Male | 17 | 16 | 17 | 18 | 45 | 61 |
| Female | 16 | 16 | 19 | 18 | 43 | 62 |

Ctrl, control; Veh, vehicle.

**Table 3. Sex distribution of animals in the five subgroups**

|  | Control | | | | |  | SE | | | | |
| --- | --- | --- | --- | --- | --- | --- | --- | --- | --- | --- | --- |
|  | Mid | Pro | Dex | Esk | Sev |  | Mid | Pro | Dex | Esk | Sev |
| Male | 9 | 9 | 10 | 10 | 7 |  | 16 | 11 | 12 | 13 | 9 |
| Female | 10 | 9 | 8 | 7 | 9 |  | 13 | 13 | 10 | 14 | 12 |

Mid, midazolam; Pro, propofol; Dex, dexmedetomidine; Esk, esketamine; Sev, sevoflurane.

**Table 4. The mean doses of intravenous anesthetics to achieve SE termination**

|  | SE+Mid  (n = 25) | SE+Pro  (n = 22) | SE+Dex  (n = 21) | SE+Esk  (n = 21) |
| --- | --- | --- | --- | --- |
| Total doses to achieve SE termination | 21.36 ± 5.22 mg/kg | 21.14 ± 3.43 mg/kg | 81.05 ± 28.65  μg/kg | 59.05 ± 13.38 mg/kg |

Mid, midazolam; Pro, propofol; Dex, dexmedetomidine; Esk, esketamine; Sev, sevoflurane.

**Table 5. Parameters of arterial blood gas analysis 5 hours after GAs administrations**

|  | SE+Veh  (n = 4) | SE+Mid  (n = 4) | SE+Pro  (n = 4) | SE+Dex  (n = 4) | SE+Esk  (n = 4) | SE+Sev  (n = 4) |
| --- | --- | --- | --- | --- | --- | --- |
| pH | 7.25 ± 0.08 | 7.29 ± 0.06 | 7.30 ± 0.07 | 7.31 ± 0.06 | 7.27 ± 0.13 | 7.30 ± 0.07 |
| pCO_2_ (mm Hg) | 42 ± 8 | 43 ± 4 | 55 ± 7 | 51 ± 9 | 57 ± 8 | 54 ± 6 |
| pO_2_ (mm Hg) | 138 ± 14 | 136 ± 11 | 128 ± 22 | 141 ± 18 | 126 ± 7 | 131 ± 11 |
| Glucose (mmol/L) | 7.4 ± 0.7 | 7.8 ± 0.5 | 8.0 ± 0.8 | 9.3 ± 1.6 | 8.3 ± 1.3 | 7.7 ± 1.3 |
| BE (mmol/L) | -10.6 ± 1.3 | -9.5 ± 1.5 | -7.1 ± 2.2 | -8.7 ± 1.8 | -9.2 ± 1.6 | -7.0 ± 1.7 |
| Lactate (mmol/L) | 2.6 ± 0.9 | 2.6 ± 1.3 | 4.0 ± 0.3 | 2.9 ± 0.9 | 3.2 ± 0.7 | 2.4 ± 0.8 |

pCO_2_, arterial carbon dioxide tension; pO_2_, arterial oxygen tension; BE, base excess; Veh, vehicle; Mid, midazolam; Pro, propofol; Dex, dexmedetomidine; Esk, esketamine; Sev, sevoflurane. Data were statistically analyzed by a two-way ANOVA followed by a Sidak’s test. pH: two-way-ANOVA_sex x group_*F*_(5,12)_ = 3.01, *p* = 0.055; two-way-ANOVA_group_*F*_(5,12)_ = 0.42, *p* = 0.824; two-way-ANOVA_sex_*F*_(1,12)_ = 0.51, *p* = 0.491. pCO_2_: two-way-ANOVA_sex x group_*F*_(5,12)_ = 0.05, *p* = 0.998; two-way-ANOVA_group_*F*_(5,12)_ = 1.86, *p* = 0.176; two-way-ANOVA_sex_*F*_(1,12)_ = 0.02, *p* = 0.893. pO_2_: two-way-ANOVA_sex x group_*F*_(5,12)_ = 0.15, *p* = 0.975; two-way-ANOVA_group_*F*_(5,12)_ = 0.44, *p* = 0.816; two-way-ANOVA_sex_*F*_(1,12)_ = 0.23, *p* = 0.641. Glucose: two-way-ANOVA_sex x group_*F*_(5,12)_ = 0.39, *p* = 0.847; two-way-ANOVA_group_*F*_(5,12)_ = 1.22, *p* = 0.358; two-way-ANOVA_sex_*F*_(1,12)_ = 0.57, *p* = 0.465. BE: two-way-ANOVA_sex x group_*F*_(5,12)_ = 0.57, *p* = 0.719; two-way-ANOVA_group_*F*_(5,12)_ = 2.22, *p* = 0.119; two-way-ANOVA_sex_*F*_(1,12)_ = 0.002, *p* = 0.966. Lactate: two-way-ANOVA_sex x group_*F*_(5,12)_ = 1.17, *p* = 0.379; two-way-ANOVA_group_*F*_(5,12)_ = 1.76, *p* = 0.196; two-way-ANOVA_sex_*F*_(1,12)_ = 0.65, *p* = 0.437.

**Supplementary Fig. 1.** Total time of exploration in the (A) acquisition and (B) test sessions. (C) Mean swim velocity of trainings in the acquisition sessions. (D) Schematic diagram of the visible platform test in the Morris water maze. (E) Mean path length across four trainings. Ctrl, control. Ctrl + no treatment, n = 33; SE + no treatment, n = 19; Ctrl + Veh, n = 36; SE + Veh, n = 31; Ctrl + GAs, n = 86; SE + GAs, n = 109. Error bars represent the standard error of the mean (SEM). Data were statistically analyzed by a two-way ANOVA followed by a Sidak’s test. Exploration time in the acquisition session: two-way-ANOVA_sex x group_*F*_(5,302)_ = 0.88, *p* = 0.497; two-way-ANOVA_group_*F*_(5,302)_ = 1.52, *p* = 0.185; two-way-ANOVA_sex_*F*_(1,302)_ = 2.32, *p* = 0.128. Exploration time in the test session: two-way-ANOVA_sex x group_*F*_(5,302)_ = 0.98, *p* = 0.432; two-way-ANOVA_group_*F*_(5,302)_ = 1.97, *p* = 0.083; two-way-ANOVA_sex_*F*_(1,302)_ = 0.17, *p* = 0.677. Mean swim velocity: two-way-ANOVA_sex x group_*F*_(5,302)_ = 0.56, *p* = 0.732; two-way-ANOVA_group_*F*_(5,302)_ = 3.85, *p* = 0.002; two-way-ANOVA_sex_*F*_(1,302)_ = 0.03, *p* = 0.872. Mean path length in the visible platform of water maze: two-way-ANOVA_sex x group_*F*_(5,302)_ = 2.36, *p* = 0.052; two-way-ANOVA_group_*F*_(5,302)_ = 1.77, *p* = 0.119; two-way-ANOVA_sex_*F*_(1,302)_ = 0.16, *p* = 0.694.

**Supplementary Fig. 2.** Effects of sex on (A) SE duration, (B) latency to SE termination, (C) mortality, and (D-J) a series of behavioural parameters. Ctrl, control; Veh, vehicle. Male, n = 159; Female, n =155. Error bars represent the standard error of the mean (SEM). Data were statistically analyzed by a two-way ANOVA or Scheirer-Ray-Hare test followed by a Sidak’s test or by the Fisher’s exact test. SE duration: two-way-ANOVA_sex_*F*_(1,302)_ = 1.56, *p* = 0.213. Latency to SE termination: two-way-ANOVA_sex_*F*_(1,254)_ = 1.68, *p* = 0.196. Distance traveled: two-way-ANOVA_sex_*F*_(1,302)_ = 1.69, *p* = 0.194. Rearing: two-way-ANOVA_sex_*F*_(1,302)_ = 0.0004, *p* = 0.984. Time in the center: two-way-ANOVA_sex_*F*_(1,302)_ = 1.68, *p* = 0.196. Discrimination index: two-way-ANOVA_sex_*F*_(1,302)_ = 0.99, *p* = 0.321. Mean path length: two-way-ANOVA_sex_*F*_(1,302)_ = 2.34, *p* = 0.127. Time in the target quadrant: two-way-ANOVA_sex_*F*_(1,302)_ = 1.90, *p* = 0.169. Platform crossing: two-way-ANOVA_sex_*F*_(1,302)_ = 0.99, *p* = 0.320.

1.	Tetz LM, Rezk PE, Ratcliffe RH, Gordon RK, Steele KE, Nambiar MP. Development of a rat pilocarpine model of seizure/status epilepticus that mimics chemical warfare nerve agent exposure. Toxicol Ind Health. 2006;22(6):255-66.

2.	De Araujo Furtado M, Aroniadou-Anderjaska V, Figueiredo TH, Apland JP, Braga MFM. Electroencephalographic analysis in soman-exposed 21-day-old rats and the effects of midazolam or LY293558 with caramiphen. Ann N Y Acad Sci. 2020 Nov;1479(1):122-33.
